# Low-Temperature Performance Enhancement of Warm Mix Asphalt Binders Using SBS and Sasobit: Towards Durable and Green Pavements

**DOI:** 10.3390/ma18204756

**Published:** 2025-10-17

**Authors:** Xuemao Feng, Mingchen Li, Yifu Meng, Jianwei Sheng, Yining Zhang, Liping Liu

**Affiliations:** 1 Guangxi New Development Transportation Group Co., Nanning 530029, China; xfzfxm1984@163.com; 2 School of Civil Engineering, Central South University, Changsha 410083, China; 3 School of Infrastructure Engineering, Dalian University of Technology, Dalian 116024, China; 4 The Key Laboratory of Road and Traffic Engineering, Ministry of Education, Tongji University, Shanghai 201804, China; 2110767@tongji.edu.cn (Y.M.);

**Keywords:** SBS modified asphalt, Sasobit, low temperature performance, fatigue performance, crumb rubber, aromatic oil

## Abstract

With growing emphasis on environmental protection and sustainability in highway construction, the high mixing and compaction temperatures of styrene-butadiene-styrene (SBS)-modified asphalt have raised concerns regarding energy consumption and pollutant emissions. Sasobit, a warm-mix additive with a melting point of 99 °C, effectively reduces asphalt viscosity and construction temperatures while enhancing high-temperature performance; however, it may adversely affect low-temperature crack resistance. To address this challenge, this study developed low-dosage Sasobit–SBS composite asphalt incorporating aromatic oil and crumb rubber to reduce production temperatures while maintaining performance. Evaluations on binder properties and mixture performance showed that Sasobit effectively lowers mixing temperatures and preserves rutting resistance, while external modifiers, especially crumb rubber, significantly enhance low-temperature crack resistance (by 24%) and fatigue life (by 50%). Moreover, the crumb rubber formulation reduced production costs by 11% compared to conventional SBS asphalt, demonstrating a practical and cost-effective strategy for improving durability in cold regions.

## 1. Introduction

Asphalt pavements account for a significant proportion of high-grade highways in China. In recent years, the continuous increase in traffic volume, coupled with widespread issues of overloading, has imposed increasingly stringent performance requirements on asphalt pavements [1,2]. Improving the performance and extending the service life of asphalt pavements has therefore become a pressing concern in the field of road engineering. Among the many factors influencing pavement durability, the intrinsic properties of asphalt materials play a crucial role [3,4,5].

Currently, unmodified asphalt remains the most widely used binder in Chinese road construction. However, its inherent shortcomings—such as poor thermal stability and susceptibility to oxidative aging—often lead to premature pavement distresses, including cracking and rutting [6,7,8,9]. These deteriorations significantly compromise pavement service life, particularly under the high loads and channelized traffic conditions characteristic of modern highways in China. Accordingly, improving the performance of asphalt binders has become an essential strategy for achieving longer-lasting pavements.

To address these challenges, polymer-modified asphalts—most notably those modified with styrene-butadiene-styrene (SBS)—have gained widespread application due to their superior mechanical properties and enhanced durability [10,11,12]. Nevertheless, the high construction temperatures (typically around 175 °C) required for SBS-modified asphalt introduce new challenges [13,14]. These include elevated energy consumption during mixing and paving, increased emissions of harmful gases such as SO_2_, CO, and NO_x_, and accelerated binder aging due to prolonged exposure to high temperatures [15,16,17]. These issues conflict with growing environmental concerns and the need for sustainable infrastructure solutions. As a result, considerable research has focused on developing warm-mix asphalt (WMA) technologies, which aim to reduce production temperatures while maintaining or even improving performance. Among various WMA additives, Sasobit—a long-chain aliphatic hydrocarbon wax with a melting point of approximately 99 —has received particular attention [18,19,20,21]. Sasobit significantly reduces the high-temperature viscosity of asphalt binders, allowing for lower mixing and compaction temperatures, thereby contributing to energy savings and emission reductions [22,23]. Experimental results reveal that, regardless of aggregate type or source, the addition of 1% Sasobit reduces heating energy demand by around 2.8% and CO_2_ emissions by approximately 3.0% [24]. In addition to its role as a WMA additive, Sasobit has also demonstrated the ability to enhance high-temperature performance and rutting resistance when used as a modifier. However, a growing body of evidence indicates that Sasobit adversely affects the low-temperature performance of asphalt binders, increasing the risk of thermal cracking in cold climates [25,26,27,28]. Liu et al. [29] evaluated the impact of Sasobit incorporation on the low-temperature performance of asphalt binders through a series of tests, including the Bending Beam Rheometer (BBR), Direct Tension Test (DTT), Asphalt Binder Cracking Device (ABCD), and Indirect Tensile Test (IDT). The results demonstrated that the addition of Sasobit led to an increase in the cracking temperature of the asphalt binders, with the effect becoming more pronounced as the Sasobit content increased. Similarly, Wu et al. [30] found that although incorporating Sasobit into SBS-modified asphalt could reduce the mixing temperature by 16 °C, it also resulted in a significant reduction in low-temperature ductility. On the other hand, SBS modification is known for its beneficial effects on both elasticity and low-temperature cracking resistance, although it typically requires higher processing temperatures.

Given the complementary characteristics of SBS and Sasobit, a composite modification approach that integrates both additives holds significant promise. This study aimed to optimize the proportions of raw materials and the preparation process of Sasobit–SBS composite-modified asphalt to achieve both a reduction in mixing temperature and the preservation of the composite-modified asphalt’s low-temperature performance. For this purpose, four types of Sasobit–SBS composite-modified asphalts with varying raw material ratios were produced in this study. Subsequently, their high-temperature performance, low-temperature performance, and aging resistance were evaluated through Dynamic Shear Rheometer (DSR), Bending Beam Rheometer (BBR), and Gel Permeation Chromatography (GPC) tests. Furthermore, the performance of the corresponding asphalt mixtures was comparatively assessed using rutting tests, freeze–thaw splitting tests, low-temperature semicircular bending tests, and indirect tensile fatigue tests. Ultimately, an optimal composite-modified asphalt formulation was proposed to address the existing challenges related to the insufficient low-temperature performance of Sasobit–SBS-modified asphalt. The findings of this study are expected to contribute to reducing the cost of Sasobit–SBS composite-modified asphalt and to facilitate its wider application in cold-region pavements.

## 2. Materials and Methods

### 2.1. Materials

#### 2.1.1. Asphalt

In the study, modified asphalt was prepared using unmodified asphalt with a penetration grade of 70, and its basic properties are shown in Table 1.

#### 2.1.2. Additives

**Figure 1:**
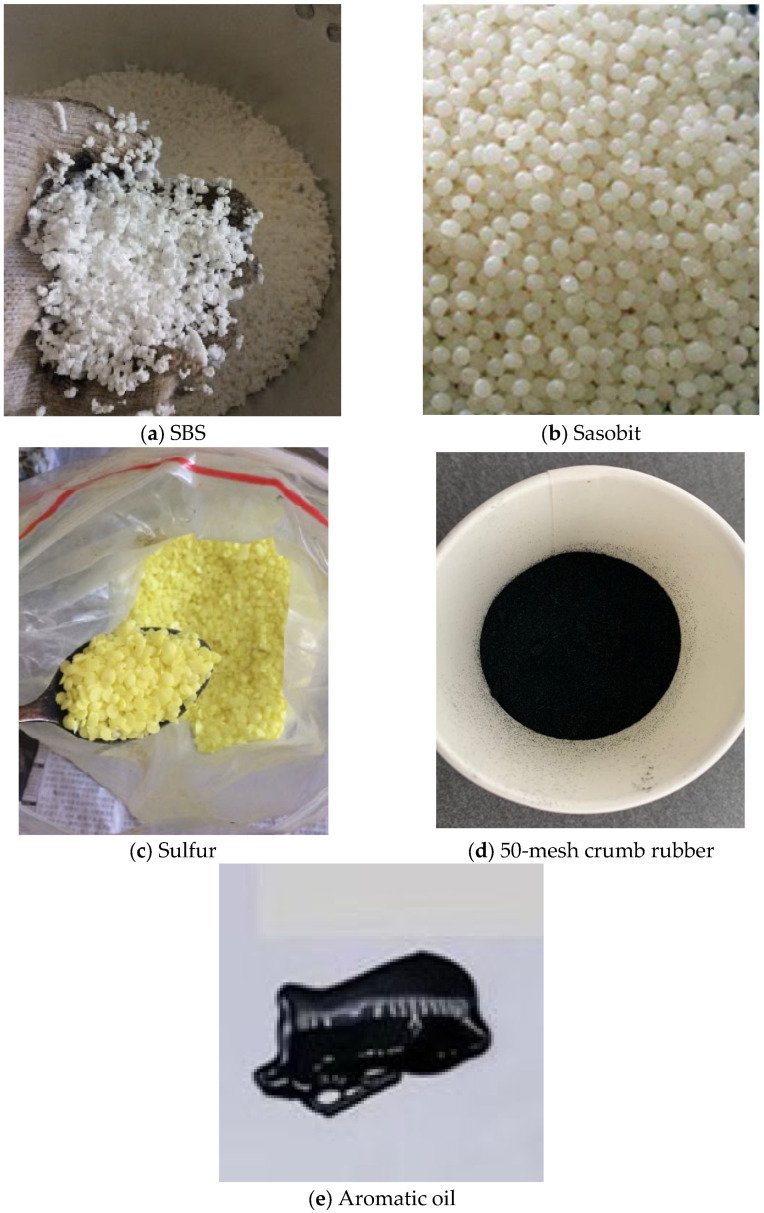
The additives used in this study.

To prepare Sasobit–SBS composite-modified asphalt and enhance its low-temperature crack resistance, five additives were used in this study, namely Sasobit, SBS, sulfur, crumb rubber, and aromatic oil, as shown in Figure 1.

#### 2.1.3. Preparation Process of the Sasobit–SBS-Modified Asphalt

This study aims to reduce the SBS modifier dosage through the incorporation of Sasobit, while simultaneously enhancing the low-temperature crack resistance of the composite-modified asphalt. Accordingly, four modified asphalt formulations with varying compositions were prepared. It should be noted that the dosages of modifiers used in this study were all referenced from the existing literature [31,32]. It should be noted that, according to the asphalt viscosity test results, formulations B, C, and D can reduce the mixing temperature of SBS-modified asphalt mixtures by approximately 10 °C.

A: Unmodified asphalt + 4% SBS modifier + 1.5% sulfur;
B: Unmodified asphalt + 2.5% SBS Modifier + 1% Sasobit + 0.8% Aromatic oil + 1.5% sulfur;
C: Unmodified asphalt + 2.5% SBS Modifier + 1% Sasobit + 18% 50-mesh rubber powder + 1.5% sulfur;
D: Unmodified asphalt + 3.5% SBS + 2% Sasobit + 1.5% sulfur.

Owing to the variations in material proportions, the four formulations were produced using distinct preparation procedures. For formulation A and D, the unmodified asphalt was first heated to 165 °C until it reached a fluid state, after which the specified SBS modifier was added and sheared at high speed for 2 h. Subsequently, sulfur was incorporated, and the mixture was stirred for an additional 20 min, yielding the modified asphalt for formulation A. For formulation D, Sasobit was further added to the above mixture and stirred for another 20 min to obtain the final product.

For formulation B, the sequence in which aromatic oil is added significantly influences the final properties of the modified asphalt. Experimental results confirmed that incorporating aromatic oil at the initial stage of the preparation process produces the most favorable outcomes. The preparation of the modified asphalt began by adding aromatic oil to the base asphalt, followed by temperature control between 180 and 190 °C. A high-speed shear mixer was then activated to shear the mixture at 180–200 °C. SBS particles were introduced and sheared at high speed for 2 h until fully dissolved in the base asphalt. Subsequently, sulfur particles were added, and shearing continued for another 1.5 h while maintaining the temperature between 180 and 190 °C. Finally, Sasobit was incorporated, and the sample was sheared continuously for 20 min to complete the preparation.

Formulation C considers the addition of an appropriate amount of crumb rubber to improve the low-temperature performance of Sasobit–SBS-modified asphalt. In terms of preparation process, a high-speed shearing combined with mechanical stirring method was selected to produce the modified asphalt. The modified asphalt was prepared by first adding SBS to the base asphalt and maintaining the temperature between 180 and 190 °C. The mixture was subjected to high-speed shearing for 40 min, after which crumb rubber was added and the shearing and mixing continued for another 40 min. The high-speed shear mixer was then replaced with a mechanical stirrer, and the sample was stirred continuously for 1 h. Once a uniform mixture was achieved, sulfur particles were incorporated and stirred for an additional 20 min. Finally, Sasobit was added, and the sample was stirred continuously for a further 20 min to complete the preparation.

#### 2.1.4. Mixture Gradation Design

**Figure 2:**
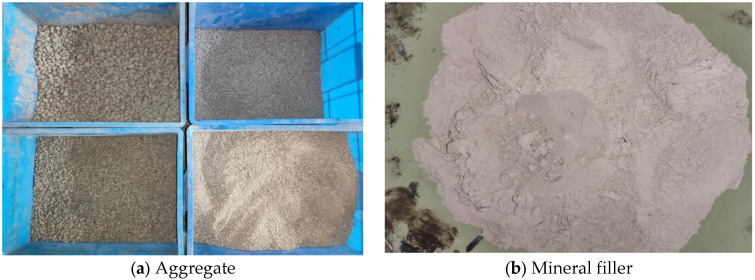
Mineral aggregate used in the study.

For each prepared modified asphalt formulation, corresponding mixtures were produced to evaluate the high-temperature performance, low-temperature crack resistance, moisture resistance, and fatigue performance of the composite-modified asphalt mixtures. The mixtures were designed using a dense-graded AC-13 gradation. The detailed gradation results are shown in Table 2. Aggregates were divided into four size categories: 0–3 mm, 3–5 mm, 5–10 mm, and 10–15 mm. Basalt was used as the coarse aggregate, limestone as the fine aggregate, and limestone powder as the mineral filler, as illustrated in Figure 2. The mixtures were designed using the Marshall method, resulting in optimum asphalt contents of 3.98%, 4.08%, 4.67%, and 4.03% for the four formulations, respectively.

### 2.2. Methods

In this study, the DSR, MSCR, BBR, and GPC tests were employed to evaluate the performance of the composite-modified asphalt, while the rutting test, low-temperature semicircular bending test, and freeze–thaw splitting test were used to assess the performance of the composite-modified asphalt mixtures.

#### 2.2.1. DSR Test

The rheological behavior of the Sasobit–SBS-modified asphalt was characterized using a DSR(AR1500ex, TA Instruments, New Castle, DE, USA). A time sweep procedure was applied to determine the complex shear modulus (G*) and phase angle (δ) over a range of temperatures. For each formulation, two specimens were tested, and the mean values were recorded. Following the AASHTO T 315 specification, measurements were carried out at test temperatures of 58 °C, 64 °C, 70 °C, and 76 °C. Four replicate specimens were prepared for each sample group in the DSR test.

#### 2.2.2. MSCR Test

The MSCR test was employed to evaluate the high-temperature performance and elastic recovery capacity of the Sasobit–SBS-modified asphalt. Growing evidence in recent studies suggests that the non-recoverable creep compliance at a stress level of 3.2 kPa (J_nr3.2_) offers a more reliable indicator of asphalt’s resistance to rutting and deformation than the conventional rutting factor-based temperature grading method. In this study, MSCR measurements were performed at 58 °C, 64 °C, 70 °C, and 76 °C. For each asphalt formulation, two specimens were tested, and the mean results were recorded. Following the AASHTO T 350 protocol, a modified calculation approach was applied to determine both J_nr3.2_ and the corresponding percent recovery (R_3.2_) under a stress of 3.2 kPa. Four replicate specimens were prepared for each sample group in the MSCR test.

#### 2.2.3. BBR Test

The low-temperature performance of the Sasobit–SBS-modified asphalt was assessed using the BBR test. Following the ASTM D6648 [33] specification, measurements were carried out at test temperatures of −12 °C and −18 °C. From the results, the creep stiffness (S) and the slope of the creep curve (m) were determined for both the SBS-modified asphalt and the Sasobit–SBS-modified binders. Each formulation was tested in duplicate, and the reported values represent the average of the two measurements. Six replicate specimens were prepared for each sample group in the BBR test.

#### 2.2.4. GPC Test

The anti-aging properties of the composite-modified asphalt were analyzed using the GPC test. It offers an effective approach for determining the molecular weight and its distribution in polymeric materials and is extensively used to study asphalt aging behavior and the blending state between virgin and aged binders, as aged binders typically exhibit a higher large molecular size (LMS) content. In this study, GPC was employed to characterize the molecular weight distribution of the prepared asphalt. The procedure involved dissolving the asphalt sample in tetrahydrofuran (THF), injecting 1 mL of the asphalt–THF solution into the instrument, recording the chromatogram showing the variation in refractive index with elution time, and calculating the weight-average molecular weight using Equation (1) [34]. Three replicate specimens were prepared for each sample group in the GPC test.

(1) $$\bar{M_{w}}=\frac{w_{i}M_{i}}{w_{i}}$$

where $\bar{M_{w}}$ represents weight-average molecular weight and *w_i_* represents the weight corresponding to molecular weight *M_i_*.

#### 2.2.5. High-Temperature Rutting Test

To evaluate the high-temperature rutting resistance of the Sasobit–SBS composite-modified asphalt mixtures, a rutting test was conducted to assess their high temperature stability. After mixing at the designed asphalt content, asphalt mixture specimens were compacted into rutting slabs measuring 300 mm × 300 mm × 100 mm using a wheel compactor. The specimens were then conditioned in a constant temperature chamber at 60 °C. During testing, the contact pressure between the loading wheel and the rutting slab was controlled at 0.7 MPa ± 0.05 MPa. Following a 48 h curing period, the rutting test was performed. The primary indicators for evaluating the high-temperature stability of the asphalt mixtures in the rutting test are the dynamic stability (DS). Lower DS values correspond to greater deformation, indicating deeper rutting and poorer high-temperature performance. Three replicate specimens were prepared for each sample group in the high-temperature rutting test.

#### 2.2.6. Low-Temperature SCB Test

In this study, the SCB test was employed to assess the low-temperature performance of Sasobit–SBS composite-modified asphalt mixtures. After preparation, the compacted mixtures were cut into semi-circular specimens. These specimens were placed on supports with a predetermined span length, and a universal testing machine (UTM-30) applied a vertical load at the midpoint of the span at a constant rate. Upon reaching a load of 10 N, the data acquisition system was reset to zero to establish the baseline. Loading continued until specimen failure, which was defined by a load drop below 0.1 kN, at which point the test was terminated. Load versus mid-span deflection data were recorded and stored via the computer connected to the testing apparatus. The primary parameter used to evaluate low-temperature performance in the SCB test is the fracture energy (G_f_), calculated by integrating the area under the load–displacement curve. A higher fracture energy value corresponds to improved resistance to low-temperature cracking. Six replicate specimens were prepared for each sample group in the SCB test.

#### 2.2.7. Freeze–Thaw Splitting Test

In this study, the freeze–thaw splitting test was employed to evaluate the moisture susceptibility of the asphalt mixtures. After preparing mixtures at their optimum asphalt contents, specimens were compacted using the Marshall hammer in accordance with the relevant standards, applying 50 blows on each face to form the test specimens. The specimens were then divided into two groups, one subjected to freeze–thaw conditioning cycles and the other kept unconditioned. Subsequently, indirect tensile strength (ITS) tests were conducted on both groups. The moisture resistance of the asphalt mixtures was quantified by the tensile strength ratio (TSR), defined as the ratio of the ITS value of the conditioned specimens to that of the unconditioned specimens. A higher TSR indicates superior moisture resistance of the asphalt mixture.

#### 2.2.8. Indirect Tensile Fatigue Test

The fatigue performance of the asphalt mixtures was evaluated using the indirect tensile fatigue test. Cylindrical specimens were prepared by cutting samples from rotationally compacted asphalt mixtures. A radial load was applied to each specimen to induce uniform tensile stress along the vertical direction inside the sample. The test was conducted on a universal testing machine at 15 °C under a stress-controlled loading mode. A continuous half-sine wave load was applied at a frequency of 10 Hz, with a stress ratio of 0.4. Horizontal deformation of the specimen was monitored and calibrated using displacement sensors. The variation in the stiffness modulus with the number of loading cycles was recorded to assess the fatigue behavior of different Sasobit–SBS composite-modified asphalt mixtures. The stiffness modulus at splitting failure was calculated using the following Equation (2) [35]. Four replicate specimens were prepared for each sample group in fatigue test.

(2) $$S=\frac{P\times \left(\nu +0.27\right)}{xt}$$

where *S* represents the stiffness of the mixture, MPa; *P* represents the vertical load peak value, N; *ν* represents the Poisson’s ratio; *x* represents the specimen horizontal radial deformation amplitude, mm; and *t* represent the average height of the specimen, mm.

## 3. Results

### 3.1. Comparison of Composite-Modified Asphalt Performance

#### 3.1.1. Rheological Properties of Modified Asphalt

**Figure 3:**
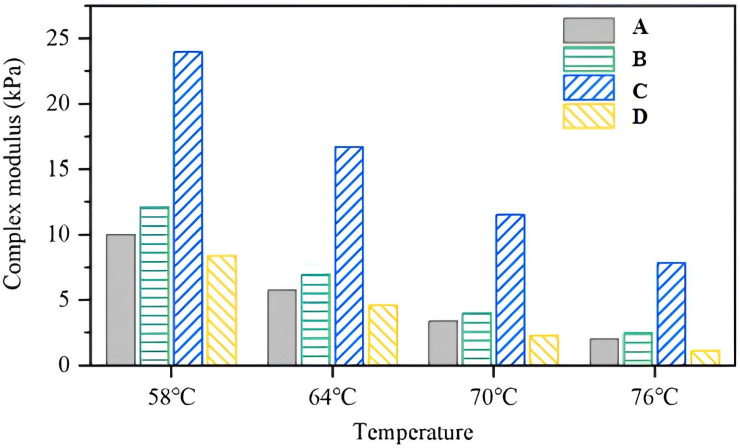
Complex shear modulus of prepared composite-modified asphalts.

The complex modulus (G*) results obtained from the DSR time sweep test are presented in Figure 3. As the temperature increased, the complex modulus of all asphalt formulations decreased, with the rate of decline gradually slowing and the differences in modulus between temperature intervals becoming less pronounced. As can be seen from the figure, among the four formulations, the complex modulus of formulation C is the least sensitive to temperature changes, indicating superior performance at both high and intermediate temperature ranges.

It can also be concluded from the figure that when comparing the complex modulus of different asphalt formulations, formulation C exhibited a significantly higher complex modulus. This indicates that the addition of rubber powder is more effective in enhancing asphalt stiffness than adding Sasobit alone. Additionally, the incorporation of aromatic oil can also enhance the complex modulus of the composite-modified asphalt, although its effect is less pronounced compared to that of rubber powder. Since the complex modulus directly reflects the mechanical properties of asphalt materials, formulation C demonstrates superior mechanical performance compared to conventional SBS-modified asphalt.

The significant increase in the complex modulus of the composite-modified asphalt containing both rubber powder and Sasobit, compared to that with Sasobit alone, can be attributed to the synergistic effects of these additives on the asphalt’s microstructure and mechanical behavior. Based on the existing studies, SEM observations from previous studies show that rubber powder is uniformly dispersed in the asphalt as swollen punctate and flocculent structures, forming a strong interface with the base asphalt [36]. This indicates that the rubber particles are well integrated into the asphalt matrix, contributing to local stiffness enhancement. FTIR analysis further reveals the presence of chemical or physical interactions between the rubber and asphalt, such as bonding or slight cross-linking, which can facilitate stress transfer and energy dissipation within the binder [37]. Beyond these microstructural effects, rubber powder also participates in forming a three-dimensional interpenetrating network with the SBS polymer during mixing and shearing processes. This network improves the binder’s elasticity, resilience, and resistance to deformation under load, thereby collectively enhancing mechanical properties such as the complex modulus.

Therefore, the combined use of rubber powder and Sasobit results in a composite-modified asphalt with enhanced stiffness and elastic response, leading to a significantly higher complex modulus than that achieved by adding Sasobit alone. This synergistic reinforcement makes the binder more resistant to deformation and improves its high-temperature performance.

#### 3.1.2. Creep and Recovery Properties of Modified Asphalt

**Figure 4:**
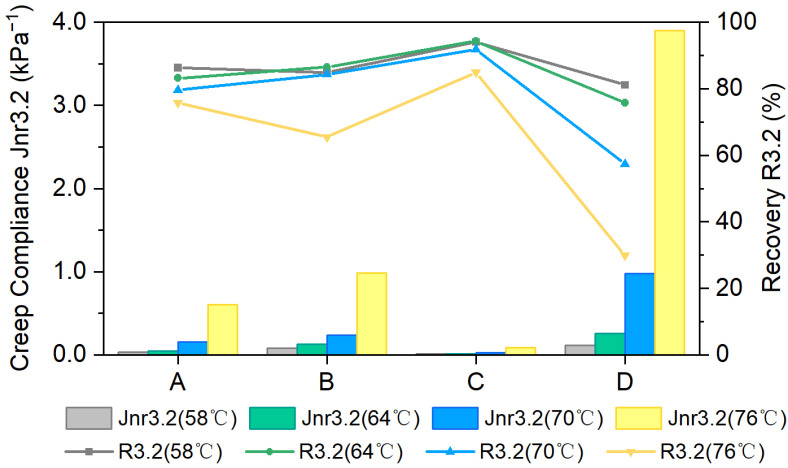
R_3.2_ and J_nr3.2_ of prepared composite-modified asphalts.

In this study, MSCR tests were conducted on various composite-modified asphalt formulations to evaluate their high-temperature performance. All samples were conditioned using the Rolling Thin-Film Oven Test (RTFOT) method. A modified MSCR calculation method was employed to determine the percent recovery (R_3.2_) and non-recoverable creep compliance (J_nr3.2_) of the SBS-modified asphalt at a stress level of 3.2 kPa. The related test results are summarized in Figure 4.

With increasing temperature, the J_nr3.2_ increased, reflecting greater viscous behavior, diminished resistance to permanent deformation, and a higher likelihood of rutting. At any given temperature, the rutting resistance of the formulations followed the order of best to worst as formulation C, followed by formulation A, then formulation B, and finally formulation D, with formulation C demonstrating the superior rutting performance. This underscores the important contribution of rubber powder to enhancing rut resistance, especially in formulation B. At the same time, comparison of J_nr3.2_ variations across different temperatures reveals that formulation C, the rubber powder composite-modified asphalt, exhibits the lowest temperature sensitivity. This indicates that it maintains strong resistance to high-temperature deformation throughout the tested temperature range, consistent with the trends observed in the complex modulus.

Also, the R_3.2_ of all composite-modified asphalts declined with increasing temperature, mainly due to shifts in the balance of viscoelastic components. Compared to formulation A, the formulations containing Sasobit showed more pronounced variations, suggesting that Sasobit enhances the temperature sensitivity of the asphalt binder.

In summary, according to the time sweep and MSCR test, incorporating either aromatic oil or rubber powder can markedly enhance the high-temperature performance of SBS-Sasobit modified asphalt.

#### 3.1.3. Low-Temperature Properties of Modified Asphalt

**Figure 5:**
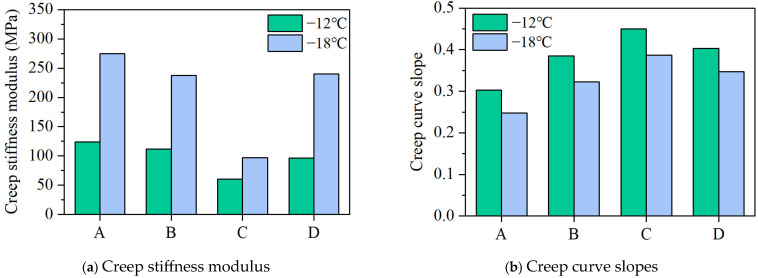
Results of BBR tests at −12 and −18 °C.

Under normal conditions, higher asphalt stiffness leads to greater internal thermal stress and reduced low-temperature performance. In the Bending Beam Rheometer (BBR) test, the creep rate (*m*) reflects the asphalt’s stress relaxation capacity, with a higher creep rate indicating a stronger ability to relieve thermal stress under low-temperature conditions. According to the SHRP study, the creep stiffness (*S*) and creep rate (*m*) at 60 s are considered key control parameters for evaluating low-temperature performance. The results of the BBR test are presented in Figure 5.

At both −12 °C and −18 °C, the general trends for creep stiffness and creep rate were as follows: C_S_ < D_S_ ≈ B_S_ < A_S_ < 300 MPa and C_m_ > D_m_ > B_m_ > A_m_ > 0.3, indicating that the stiffness and stress relaxation properties of all formulations met the SHRP recommended values. A comprehensive comparison revealed that the aromatic oil-containing formulation B was graded PG-22, while formulations C and D were graded PG-28, with the rubber powder-containing formulation exhibiting the best low-temperature performance.

Incorporating rubber powder enhances the low-temperature performance of SBS–Sasobit composite-modified asphalt while allowing for a reduction in the amount of SBS modifier required. The improvement in low-temperature performance of SBS–Sasobit composite-modified asphalt with reduced SBS content upon the addition of rubber powder can be attributed to its elastic and flexible nature, which enhances stress relaxation and mitigates thermal contraction cracking. Rubber powder forms a partial interpenetrating network with SBS, improving elasticity even at lower SBS dosages, while its porous surface structure enhances compatibility with the asphalt matrix. Moreover, it counteracts the brittleness induced by Sasobit crystallization, thereby maintaining a balanced stiffness–flexibility profile under low-temperature conditions.

#### 3.1.4. Aging Resistance of Modified Asphalt

**Figure 6:**
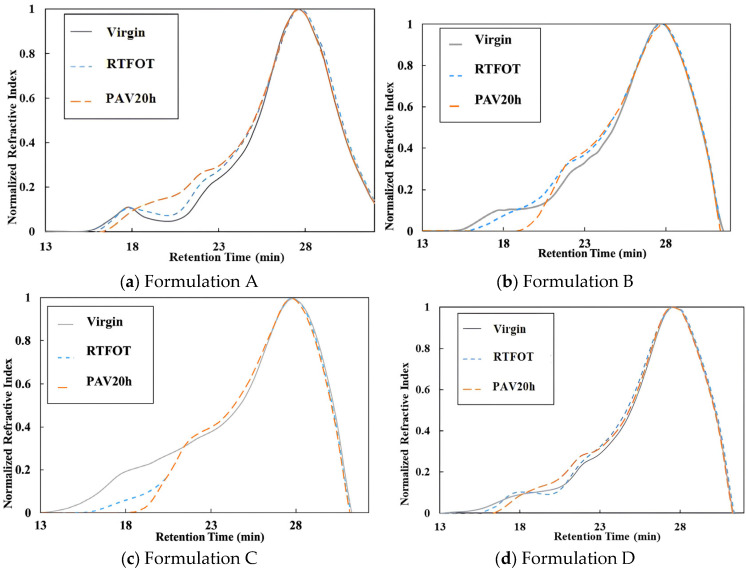
Chromatograms of asphalts at different aging states.

GPC provides an effective means to monitor polymer degradation during the aging process of polymer-modified asphalt, while also revealing the growth of high-molecular-weight fractions in the base asphalt. Variations in molecular weight at the microscopic scale across different aging stages reflect changes in the composition of the asphalt system. Accordingly, GPC analyses were conducted on original, RTFOT-aged, and 20 h PAV-aged SBS-modified asphalt, as well as on various composite-modified asphalt formulations. The resulting chromatograms are presented in Figure 6.

It can be concluded from the figure that, as aging progresses, the chromatograms of SBS-modified asphalt and various Sasobit–SBS composite-modified asphalt formulations exhibit a pronounced rightward shift in the 14~19 min region corresponding to the polymer peak, indicative of polymer degradation. This shift reflects not only the occurrence of polymer breakdown but also highlights the gradual reduction in molecular weight of the polymer components, suggesting a progressive loss of structural integrity that may influence the binder’s mechanical properties. This degradation process results in the breakdown of polymer macromolecules into smaller molecular fragments, which is reflected by an increased signal intensity in the 19~24 min region associated with asphaltenes. These observations suggest that, due to the presence of SBS modifiers, the polymeric components undergo gradual molecular weight reduction during aging, demonstrating a clear transition from larger to smaller molecular sizes.

With increasing aging time, the asphaltene peak intensity of formulation B-modified asphalt gradually increases, which can be attributed to the tendency of the abundant aromatic compounds introduced by the aromatic oil to transform into heavier fractions during aging. For formulation C, the polymer peak progressively diminishes as aging advances, and under long-term aging conditions, the polymer peak in formulation B becomes nearly undetectable, indicating severe polymer degradation. In the unaged state, the chromatogram of formulation C composite-modified asphalt shows a continuous elevation in the 14–19 min polymer region, significantly higher than that of other asphalts. This phenomenon is due to the desulfurization and degradation of rubber powder, which increases the proportion of high molecular weight species within the GPC detection range, as some degraded polymer fragments dissolve into the asphalt phase. Furthermore, polymer peaks are barely observable in formulations B and C across different aging states, and formulation D also exhibits a significant decline in polymer peak intensity with increasing aging time. These findings demonstrate that the large molecular components in all three composite-modified asphalt formulations undergo progressively severe degradation with advancing aging.

**Figure 7:**
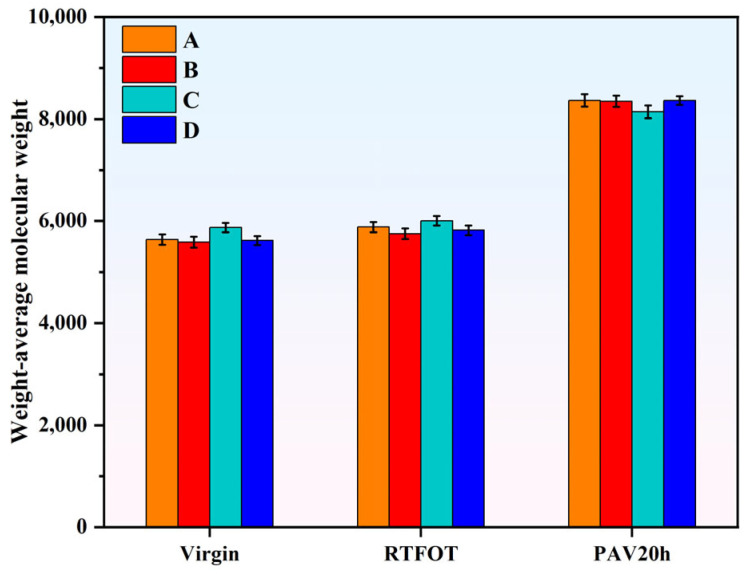
$\bar{M_{w}}$ of asphalts at different aging states.

**Figure 8:**
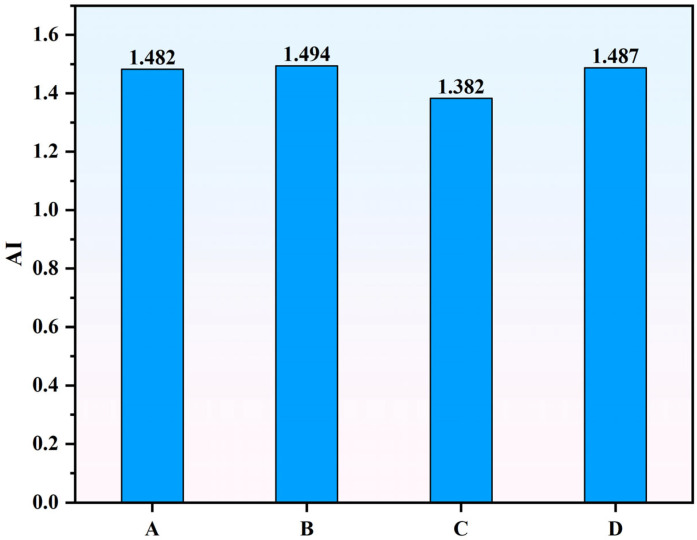
Aging resistance of asphalts at different aging states.

Furthermore, the $\bar{M_{w}}$ of the modified asphalt before and after aging were calculated via Equation (1), and the results are shown in Figure 7. The weight-average molecular weight of SBS-modified asphalt and various Sasobit–SBS composite-modified asphalt formulations continuously increases with the degree of aging. This increase in weight-average molecular weight during aging is primarily due to the oxidative aging process, which causes polymer chains and asphalt molecules to undergo oxidation and cross-linking reactions. As a result, smaller molecules combine to form larger molecular structures, leading to an overall growth in molecular weight. Additionally, some degradation products may recombine or polymerize, further contributing to the increase in large molecular fractions. Based on the $\bar{M_{w}}$, the aging resistance index (AI) of the four asphalts was calculated, defined as the ratio of $\bar{M_{w}}$ before and after aging—results are shown in Figure 8.

As shown in the figure, the AI values of formulations A, B, and D are relatively similar, while formulation C exhibits the lowest AI value. This indicates that the addition of rubber powder not only enhances the high- and low-temperature performance but also significantly improves the aging resistance of the modified asphalt.

### 3.2. Comparison of Composite-Modified Asphalt Mixture Performance

#### 3.2.1. High-Temperature Performance

**Figure 9:**
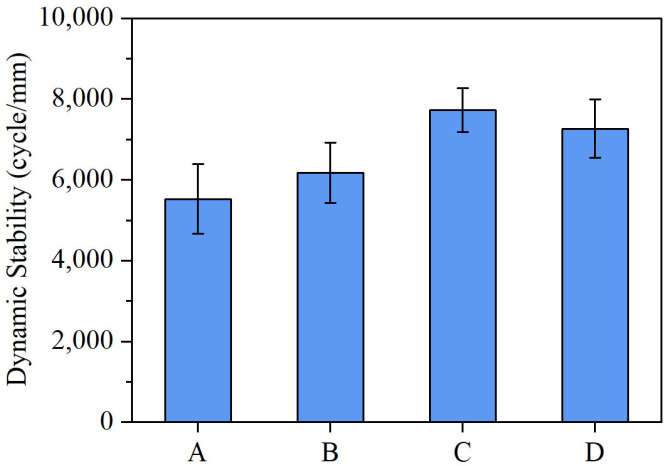
Dynamic stability of different asphalt mixtures.

The DS of different asphalt mixtures is shown in Figure 9. ANOVA statistical tests are performed based on the data to determine whether the performance differences among the samples are due to experimental error. It can be seen from the figure that compared to conventional SBS-modified asphalt mixtures, incorporating Sasobit and other additives significantly improved the dynamic stability of the mixtures. ANOVA results confirm that the asphalt formulation has a significant impact on the high-temperature performance of the asphalt mixture. The formulation with aromatic oil exhibited an 11% increase, while the rubber powder-containing mixture achieved the most pronounced enhancement, with a 40% improvement in dynamic stability. The dynamic stability of mixture D was higher than that of mixture A, indicating that the addition of Sasobit can maintain high-temperature performance while reducing the amount of SBS modifier used.

The observed improvement in dynamic stability with the addition of Sasobit and other additives can be attributed to their distinct effects on the asphalt binder’s microstructure and rheological properties. Sasobit, a wax-based additive, crystallizes within the asphalt matrix at elevated temperatures, increasing the stiffness and reducing the binder’s susceptibility to permanent deformation under load. This crystallization enhances the high-temperature performance without requiring higher SBS content. Rubber powder contributes by forming a three-dimensional elastic network within the binder, which improves elasticity and energy dissipation during loading, thereby reducing rutting potential. Aromatic oil, while primarily acting as a softening agent, optimizes the binder’s viscoelastic balance, slightly improving rutting resistance. The combined action of these modifiers results in a binder with enhanced stiffness, elasticity, and deformation resistance, leading to significantly improved dynamic stability in the modified mixtures.

#### 3.2.2. Low-Temperature Performance

**Figure 10:**
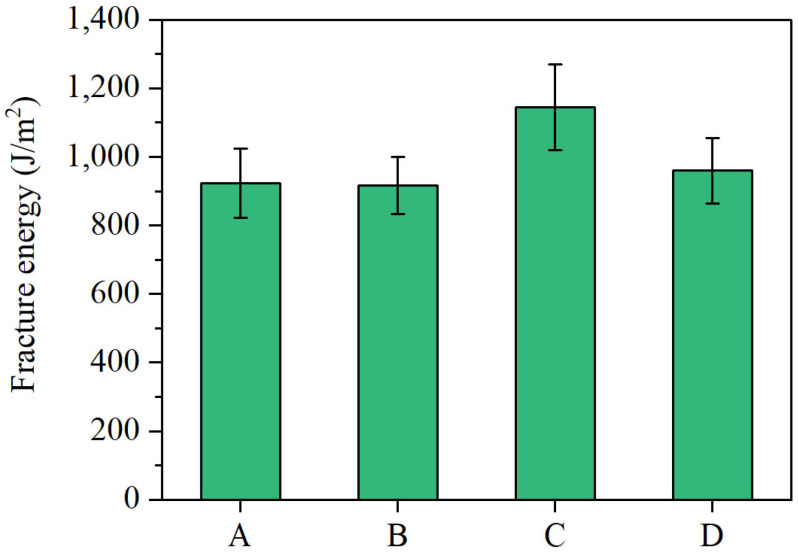
Fracture energy of different asphalt mixtures.

Figure 10 presents the results of the SCB tests for the different mixtures. Also, ANOVA statistical tests are performed based on the data to determine whether the performance differences among the samples are due to experimental error. As can be seen from the figure, by comparing the SCB fracture energy of different asphalt mixture formulations, the ranking of low-temperature performance was established as follows: Gf(C) > Gf(D) > Gf(A) > Gf(B), which is consistent with the BBR test results. Compared to conventional SBS-modified asphalt mixtures, all three Sasobit-containing composite-modified asphalt formulations demonstrated superior low-temperature performance. Notably, the formulation containing rubber powder exhibited an approximately 24% improvement in low-temperature crack resistance compared to the control group. The ANOVA statistical results also indicate that significant differences exist in the low-temperature performance among the different samples.

The superior low-temperature performance observed in the rubber powder-containing formulation (C) can be attributed to the unique reinforcing effects of rubber particles within the asphalt binder. Unlike formulations without rubber, the rubber particles introduce enhanced elasticity and flexibility, which significantly improve the binder’s ability to accommodate and relax thermal stresses during low-temperature conditions. This elastic network formed by rubber effectively reduces stress concentrations and delays crack initiation and propagation. As a result, the binder maintains greater ductility and toughness, preventing brittle failure and enhancing overall low-temperature crack resistance.

#### 3.2.3. Moisture Resistance

**Figure 11:**
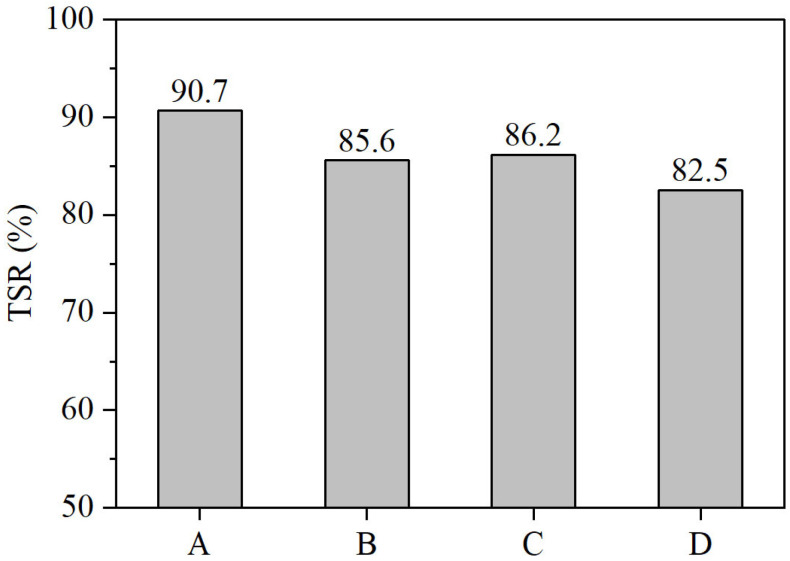
TSR of different asphalt mixtures.

The TSR of different asphalt mixtures is shown in Figure 11. It can be seen from the figure that all Sasobit–SBS composite-modified asphalt mixtures met the specification requirement for TSR (>80%), although their TSR values were slightly lower than those of the SBS-modified asphalt mixtures. Compared to the SBS mixture, the TSR of formulations B, C, and D decreased by 5%, 4% and 8%, respectively.

The reduction in moisture susceptibility caused by the addition of Sasobit can be attributed to its waxy crystalline nature, which alters the adhesive and interfacial properties between the asphalt binder and aggregates. Specifically, the crystallization of Sasobit may increase the hydrophilicity at the binder–aggregate interface, facilitating water ingress and weakening the bond strength. Additionally, Sasobit increases the stiffness and brittleness of the binder under low-temperature and moisture conditions, reducing the mixture’s toughness and resistance to stripping. Furthermore, the crystalline structure can disrupt the uniformity and continuity of the asphalt film, compromising its ability to effectively coat and protect aggregates from moisture damage. Consequently, despite improvements in high-temperature performance, Sasobit’s influence on binder microstructure can negatively affect the water stability of asphalt mixtures.

#### 3.2.4. Fatigue Performance

**Figure 12:**
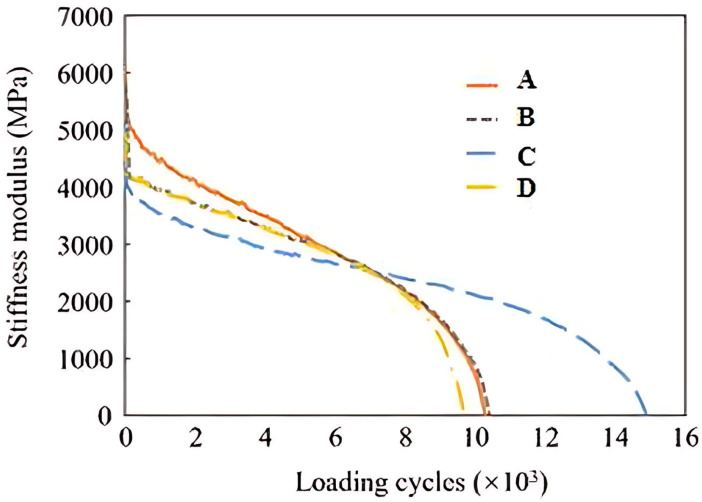
Stiffness modulus evolution of different asphalt mixtures in indirect tensile fatigue test.

This study evaluated the fatigue performance of various Sasobit–SBS-modified asphalt mixtures using indirect tensile fatigue tests. A stress-controlled loading mode with a stress ratio of 0.4 was applied. Fatigue life was defined as the number of load cycles until specimen failure. The degradation of the stiffness modulus over the loading cycles was analyzed to assess fatigue behavior. Four specimens were prepared for each asphalt type. The test results are presented in Figure 12.

By comparing the number of loading cycles at specimen failure (defined as fatigue life, Nfr) for different formulations, the general trend observed was Nfr(D) < Nfr(A) = Nfr(B) < Nfr(C). The overall fatigue performance of the Sasobit–SBS composite-modified asphalt mixtures prepared in this study was comparable to that of the SBS-modified asphalt mixtures. The shorter fatigue life of formulation D indicates that the positive effect of Sasobit on the fatigue resistance of asphalt mixtures is less significant than that of the SBS modifier. In contrast, formulation C exhibited the best fatigue resistance, highlighting the substantial performance enhancement imparted by rubber powder as an additive in the Sasobit–SBS composite-modified asphalt.

In summary, formulation C demonstrated superior performance over all other formulations in terms of both asphalt properties and asphalt mixture performance. These results suggest that incorporating crumb rubber into Sasobit–SBS-modified asphalt can substantially enhance its overall properties—particularly low-temperature cracking resistance—while simultaneously offering multiple advantages, including reduced SBS dosage, lower mixing temperatures, and improved low-temperature performance. Although the moisture sensitivity of crumb rubber-modified composite asphalt is slightly inferior to that of SBS asphalt, it still meets the specification requirements. These results highlight the promise of combining SBS and Sasobit for warm-mix asphalt binders to achieve enhanced low-temperature performance, improved durability, and greater environmental sustainability.

### 3.3. Economic Cost Analysis

Beyond the performance characteristics of the formulations, economic feasibility plays a critical role in determining their practical adoption in engineering projects. Table 3, Table 4, Table 5 and Table 6 provide a comparative analysis of the production costs associated with the four composite-modified asphalt formulations, expressed on a per-ton basis. As illustrated, formulation C stands out due to the incorporation of crumb rubber, which not only enhances its low-temperature performance but also allows for a reduction in SBS modifier content. This dual advantage contributes to formulation C achieving the lowest production cost among the evaluated options. Compared to conventional SBS-modified asphalt, its production cost is reduced by 9%. The cost-effectiveness combined with its balanced mechanical properties makes formulation C a particularly attractive candidate for large-scale engineering applications, where both performance and budget constraints must be carefully considered.

### 3.4. Discussion

Table 7 presents a comparison of the performance of different asphalt mixture formulations.

It can be concluded from the table that incorporating rubber powder into SBS- and Sasobit-modified asphalt effectively enhances both high- and low-temperature performance. However, its moisture resistance remains lower than that of conventional SBS-modified asphalt. This is mainly because Sasobit enhances the rheological properties and reduces the construction temperature of asphalt but does not improve its polarity or interfacial adhesion. On the contrary, the crystallization of Sasobit and the resulting increase in brittleness may further compromise the binder’s resistance to moisture-induced stripping. To address this issue, an anti-stripping agent can be incorporated during the preparation of the mixture. Although the incorporation of Sasobit may adversely affect the moisture resistance of asphalt mixtures, it effectively lowers the production temperature. Compared with conventional SBS-modified asphalt, the composite-modified asphalt developed in this study, containing both Sasobit and crumb rubber, can reduce the mixture production temperature by approximately 10 °C. Based on this reduction, it is estimated that the use of this modified asphalt could decrease carbon emissions by approximately 600 kg per 1000 t of asphalt mixture produced. In addition to Sasobit, foaming and zeolite technologies can also be employed in practice to reduce the mixing temperature of asphalt mixtures. Although the composite-modified asphalt proposed in this study does not achieve as large a reduction in production temperature as these two methods, it exhibits superior in-service performance. Therefore, the choice of warm-mix technology in actual projects can be made based on specific engineering requirements.

## 4. Conclusions

This study aims to develop low-dosage Sasobit–SBS composite-modified asphalt formulations that not only lower mixing and construction temperatures but also optimize both high- and low-temperature performance. To improve the formulations, additives such as aromatic oil and crumb rubber were incorporated, and comprehensive performance evaluations were conducted on both the asphalt binders and the mixtures. The main conclusions are as follows:

1. Under the tested formulations and conditions, composite modification with aromatic oil or crumb rubber was found to mitigate the inherent low-temperature deficiencies of Sasobit while maintaining its advantageous high-temperature performance. These modifications improved the balance between thermal susceptibility and aging resistance, thereby enhancing the durability of asphalt binders. Among the additives examined, crumb rubber showed the most pronounced improvement, suggesting its potential effectiveness for optimizing Sasobit-based formulations.
2. The incorporation of Sasobit and various additives into asphalt mixtures significantly improved high-temperature stability, with rutting resistance increasing by 11% to 40%. Although moisture damage resistance experienced a slight reduction, all formulations remained compliant with specification requirements. Among them, the rubber powder formulation (2.5% SBS modifier + 1% Sasobit + 18% 50-mesh crumb rubber) demonstrated the best overall performance, delivering a 24% enhancement in low-temperature crack resistance and a 50% increase in fatigue life, and is thus recommended as the optimal formulation in this study.
3. The incorporation of crumb rubber under the studied conditions not only enhanced the low-temperature performance of composite-modified asphalt but also effectively reduced the required SBS dosage, resulting in an estimated 11% reduction in production costs compared with conventional SBS-modified asphalt. While these results are promising for balancing cost and performance, further validation under broader material sources and field conditions is necessary before generalization to large-scale engineering practice.

## Figures and Tables

**Table 1:** Basic properties of the selected unmodified asphalt.

Properties	Penetration /0.1 mm	15 °C Ductility /cm	Softening Point /°C	Mass Loss After RTFOT /%	15 °C Ductility After RTFOT /cm
Result	66.4	>100	49.8	0.03	24.3

**Table 2:** Gradation of the modified asphalt mixture.

AC-13	Cumulative Percent Passing of Each Sieve/%									
	16	13.2	9.5	4.75	2.36	1.18	0.6	0.3	0.15	0.075
Upper limit	100	100	85	68	50	38	28	20	15	8
Designed gradation	100	94	84	60.2	48.7	25.2	18.4	10.2	7.5	4.4
Lower limit	100	90	68	38	24	15	10	7	5	4

**Table 3:** Production costs for formulation A.

Raw Materials	Asphalt	SBS	Sulfur
Unit price (CNY/t)	4400	15,000	2350
Dosage (kg)	945	40	15
Total price (CNY/t)	4793.25		

**Table 4:** Production costs for formulation B.

Raw Materials	Asphalt	SBS	Sasobit	Aromatic Oil	Sulfur
Unit price (CNY/t)	4400	15,000	35,000	4000	2350
Dosage (kg)	942	25	10	8	15
Total price (CNY/t)	4937.05				

**Table 5:** Production costs for formulation C.

Raw Materials	Asphalt	SBS	Sasobit	Crumb Rubber	Sulfur
Unit price (CNY/t)	4400	15,000	35,000	1000	2350
Dosage (kg)	780	25	10	180	15
Total price (CNY/t)	4372.25				

**Table 6:** Production costs for formulation D.

Raw Materials	Asphalt	SBS	Sasobit	Sulfur
Unit price (CNY/t)	4400	15,000	35,000	2350
Dosage (kg)	930	35	20	15
Total price (CNY/t)	5352.25			

**Table 7:** Comparison of the performance of different asphalt mixture formulations.

Performance	A	B	C	D
Dynamic stability (cycle/min)	5526	6176	7732	7262
Fracture energy (J/m^2^)	923	917	1144	960
TSR (%)	90.7	85.6	86.2	82.5

## Data Availability

The original contributions presented in this study are included in the article. Further inquiries can be directed to the corresponding authors.
